# A Rotational Gyroscope with a Water-Film Bearing Based on Magnetic Self-Restoring Effect

**DOI:** 10.3390/s18020415

**Published:** 2018-01-31

**Authors:** Dianzhong Chen, Xiaowei Liu, Haifeng Zhang, Hai Li, Rui Weng, Ling Li, Wanting Rong, Zhongzhao Zhang

**Affiliations:** 1 MEMS Center, Harbin Institute of Technology, Harbin 150001, China; dc2e12@163.com (D.C.); lxw@hit.edu.cn (X.L.); linglimems@hit.edu.cn (L.L.); rongwanting1@163.com (W.R.); 2 Center for Integrated Spintronic Devices, Hangzhou Dianzi University, Hangzhou 310018, China; lihai@hdu.edu.cn; 3 School of Software and Microelectronics, Harbin University of Science and Technology, Harbin 150001, China; hit00@126.com; 4 Communication Research Center, Harbin Institute of Technology, Harbin 150001, China; zzzhang@hope.hit.edu.cn

**Keywords:** gyroscope, ball-disk shaped rotor, driving scheme of brushless direct current motor (BLDCM), magnetic self-restoring effect, superhydrophobic surface (SHS), water-film bearing

## Abstract

Stable rotor levitation is a challenge for rotational gyroscopes (magnetically suspended gyroscopes (MSG) and electrostatically suspended gyroscopes (ESG)) with a ring- or disk-shaped rotor, which restricts further improvement of gyroscope performance. In addition, complicated pick-up circuits and feedback control electronics propose high requirement on fabrication technology. In the proposed gyroscope, a ball-disk shaped rotor is supported by a water-film bearing, formed by centrifugal force to deionized water at the cavity of the lower supporting pillar. Water-film bearing provides stable mechanical support, without the need for complicated electronics and control system for rotor suspension. To decrease sliding friction between the rotor ball and the water-film bearing, a supherhydrophobic surface (SHS) with nano-structures is fabricated on the rotor ball, resulting in a rated spinning speed increase of 12.4% (under the same driving current). Rotor is actuated by the driving scheme of brushless direct current motor (BLDCM). Interaction between the magnetized rotor and the magnetic-conducted stator produces a sinusoidal rotor restoring torque, amplitude of which is proportional to the rotor deflection angle inherently. Utilization of this magnetic restoring effect avoids adding of a high amplitude voltage for electrostatic feedback, which may cause air breakdown. Two differential capacitance pairs are utilized to measure input angular speeds at perpendicular directions of the rotor plane. The bias stability of the fabricated gyroscope is as low as 0.5°/h.

## 1. Introduction

Rotational gyroscopes, mainly including magnetically suspended gyroscopes (MSGs) and electrostatically suspended gyroscopes (ESGs), with a high-speed spinning rotor suspended to sense input angular speeds at the rotor plane based on precession principle, exhibits higher performance than vibratory gyroscopes, which have cross-talk between drive mode and sense mode [1,2]. Shearwood et al. developed an MSG with an Al rotor levitated by electromagnetic repulsion force from the stator plain coil in 1997 [3,4]. Shanghai Jiao Tong University has done research on MSGs for years and they proposed an MSG by the MEMS (Micro-electromechanical Systems) process on silicon substrate with levitation coils, rotation coils, torque coils and a pyrolytic graphite rotor in 2006, an MSG with a rotor levitated by magnetic force about 0.7 mm over the magnet and rotated by electrostatic force in 2008, an MSG with a permanent magnet for micro-disc rotor suspension in 2010 [5,6,7]. The Air Force Research Laboratory (Wright-Patterson Air Force Base, OH, USA) and Milli Sensor Systems and Actuators (Newton, MA, USA) designed an MSG with a wheel rotor, which has teeth-like circumference to form a multi-phase variable reluctance motor with U-shaped stator segments for rotor driving [8,9]. Beihang University developed an MSG with passive magnetic bearing and active magnetic bearing to control axial and radial motions of the rotor. Influence of control parameters (damping and stiffness) to axial and radial motions is explored through analysis and simulation [10]. An ESG with electrodes for rotor driving, rotor positioning, rotor rebalance and suspension was designed in the early 1990s by Satcon (Boston, MA, USA). The angular speed sensitivity reaches 0.01°/s at 100 Hz [11]. Tokimec and Tohoku universities developed gyroscopes with disc-shaped and ring-shaped rotors on structure glass-silicon-glass, which can sense 3-axis accelerations and 2-axis angular speeds. Rotors are fabricated by single crystal silicon through deep reactive ion etching processing [12,13,14]. Compared with the gyroscope with a disc-shaped rotor, lower voltage is required in the gyroscope with a ring-shaped rotor for rotor levitation in axial direction. Two ESGs with ring-shaped rotors reported by them reach an angle random walk of 0.15${}^{\circ}/\sqrt{h}$ and 0.085${}^{\circ}/\sqrt{h}$, respectively [13,14]. Southampton University proposed an ESG through the study of a system-level model with a micro-disk suspended by electrostatic forces [15,16,17,18,19,20]. In the ESG, suspension and spin electrodes locate above and underneath the disk-shaped rotor and position control electrodes locate at the periphery of the rotor [15,18]. Rotor disk is close-loop controlled at the null position through electrostatic feedback [19,20]. A number of MSG and ESG structures have been proposed with improved control schemes [21,22,23]. However, further improvement of measurement precision is still limited by stability of rotor levitation. To decrease complexity and cost of fabrication, the driving scheme of brushless direct current motor (BLDCM) is adopted in the proposed rotational gyroscope, which produces restoring effect to magnetic ball-disk shaped rotor inherently, instead of feedback electronics. Contactless rotor levitation in ESGs and MSGs is replaced by a water-film bearing, which provides stable rotor support with low friction to spinning motion and precessional motion. Liquid bearing with low friction in principle, instead of mechanical bearing, has been applied in rotational stages in the form of satellite distributed droplets bearing [24], ionic liquid ring bearing [25], and single droplet bearing [26]. However, satellite distributed droplets bearing has static resisting torque to rotor spinning motion, resulting from shear restoring force proportional to contact angle hysteresis [24]. Spinning speed achieved of the rotational stage with this kind of bearing is only 2400 rpm, which is low for gyroscope application. Liquid ring bearing is not suitable for devices with rotor plane deflection [25] and single droplet bearing has large rotor position uncertainty of 2° [26]. Thus, none of the reported liquid bearing for rotational stage is suitable for application in gyroscope. In the proposed gyroscope design, a new form of liquid bearing, water-film bearing, is added between the rotor ball and the ball bowl at the upper surface of the lower supporting pillar. The special design of the rotor (ball-disk shape) keeps constant thickness of the water film, thus guaranteeing stable damping characteristic for rotor spinning motion and precessional motion. To decrease friction further, a superhydrophobic surface (SHS) is fabricated on the rotor ball. Application of SHS for fluidic drag reduction has been studied and experimented recently [27,28,29,30]. The novel structure design with a water-film bearing and an SHS on the rotor ball contributes to high precision of the proposed rotational gyroscope.

Section 2 illustrates the gyroscope structure and the fabrication of SHS. Section 3 explains working principles of the device from aspects of magnetic self-restoring effect of rotor, dragging torque of water-film bearing to spinning and precessional motion, sensing principle, differential capacitance detection and signal processing. In Section 4, a spectral analysis experiment to identify cut-off frequency of the low-pass filter (LPF) in a signal processing system is introduced initially. Then, the fabricated gyroscope is placed on the rate table for performance parameters’ measurement. Afterwards, an oscillating platform is constructed and dynamic character parameters of the proposed gyroscope are confirmed by curve fitting of impulse response. Finally, Section 5 summarizes the significance of the novel device.

## 2. Structure Design

### 2.1. Mechanical Structure

**Figure 1:**
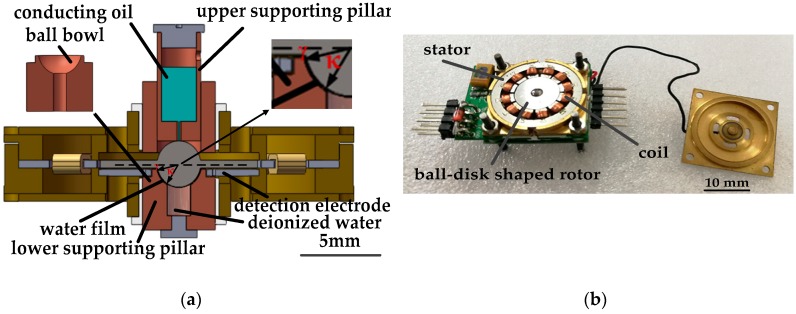
(**a**) engineering diagram of the gyroscope; (**b**) photograph of the gyroscope.

Figure 1a,b show the engineering diagram by SolidWorks (2013, Dassault Systèmes SOLIDWORKS Corp., Waltham, MA, USA) and a photograph of the proposed gyroscope. The rotor ball and the ring-shaped rotor disk are made up of stainless steel and 2J85 grade permanent magnet (FeCrCo family, remanent field is 1.3950 T, coercivity is 14 kA/m), magnetized in parallel direction, respectively. The rotor disk has an outer diameter of 10.8 mm, an inner diameter of 3 mm, and a thickness of 0.5 mm. The radius of rotor ball is 1.4 mm. In assembling, a circle belt (at the position of the great circle of the rotor ball) with a radius of 1.38 mm and a width of 0.5 mm is polished. Then, the rotor ball is glued to the rotor disk at the circle belt. The stator, with the inner radius of 5.6 mm, is designed to be of the same thickness with the rotor disk. The stator consists of silicon steel sheets and 12 poles evenly allocated with enameled wire winding around each of them. The driving scheme of BLDCM is adopted by the stator to generate a circular rotational magnetic field, which drives the magnetized rotor to a stable, at high spinning speed, with the angular momentum of *H*. With zero input angular speed (defined as zero state), the stator, the rotor disk and the electrode plate are parallel and coaxial to each other. Upper and lower supporting pillars limit the position of the rotor and three rotational degrees of freedom of the rotor ball are guaranteed. Conducting oil is filled in the cavity of the upper supporting pillar to set up a reliable electric path from the upper supporting pillar to the rotor ball. AC modulation signal *V_mod_* is added to the rotor through the electric path. Deionized water in the cavity of the lower supporting pillar forms a thin water film as a bearing between the rotor ball surface and the ball bowl when the rotor rotates at a high spinning speed. SHS is fabricated on the rotor ball to decrease sliding friction further and guarantees the stable spinning motion and precessional motion (with suitable damping characteristic) of the rotor. Air bearing will produce a much lower sliding friction to the rotor, while it is mainly used as journal bearing and is not suitable for the designed gyroscope. Another factor that prevents the application of air bearing is that such a low damp will lead to a long dynamic adjusting time to precessional motion when sensing input angular speed. A detection electrode plate is fixed by the lower supporting pillar 100 μm to the lower surface of rotor disk (at zero state), forming four tilt induced capacitors with it.

### 2.2. Fabrication of SHS on the Rotor Ball

**Figure 2:**
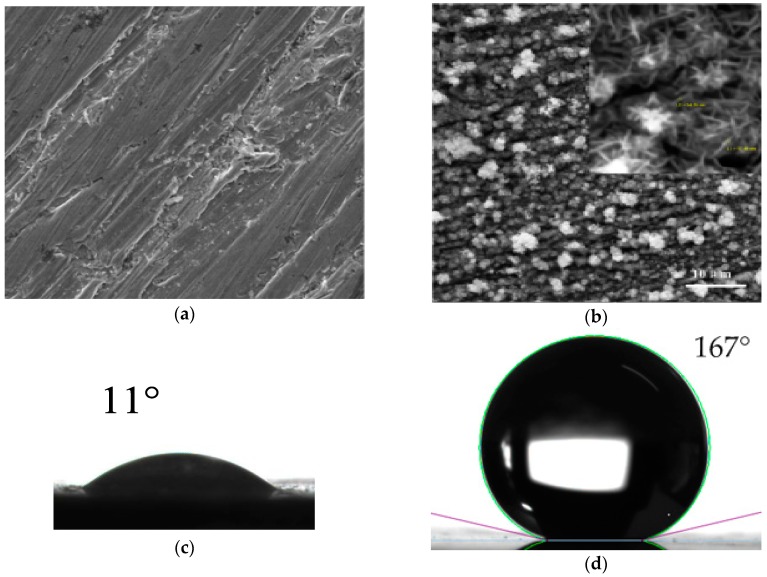
SEM images of (**a**) untreated rotor ball surface and (**b**) the rotor ball surface with fabricated nanosheets; Optical photos of a water droplet on (**c**) untreated carbon steel sheet surface and (**d**) SHS.

The carbon steel rotor ball was polished with 1700# sandpapers, cleaned ultrasonically in acetone and deionized water in sequence, and dried at room temperature. In addition, 200 mL of acid solution (mixture of hydrochloric acid (HCl) and 0.2 M potassium chloride (KCl)) was heated to 70 °C in a glass breaker. The rotor ball was immersed in it with oxygen introduced (150 sccm) to fabricate lotus-leaf-like nanostructures of Fe_3_O_4_. A magnetic rotor rotated at 120 rpm in the solution to accelerate the reaction. Afterwards, the ball was cleaned in distilled water and dried in N_2_ for 1.5 h. Finally, the ball was immersed in an ethanol solution of fluorinated silane (0.5 wt %) for half an hour and dried in a vacuum oven at 120 °C for an hour. Then, SHS was fabricated. SEM images of Fe substrate, fabricated Fe_3_O_4_ nanosheets with a small image at a higher resolution, are shown in Figure 2a,b. Nanosheets with the thickness of 50–60 nm are uniformly distributed on the ball surface (Figure 2b). To evaluate wettability of the material surface conveniently, water contact angles (CAs) of carbon steel sheet of the same material (Figure 2c) and fabricated SHS on it with the same process technology (Figure 2d) are measured. Thus, SHS with the same water CA of 167° is fabricated on a rotor ball.

Driving scheme of BLDCM is adopted in the gyroscope with six driving pairs (two opposing poles with wires winding around) producing a rotating driving magnetic field. Under the same root mean square (RMS) driving current of 110 mA/phase, steady-state spinning speed for rotor without and with SHS are 8970 rpm and 10084 rpm, respectively. Therefore, SHS results in rated angular momentum (*H*) increase of 12.4% ((10084 − 8970)/8970 = 12.4%) [31].

## 3. Operational Principle

**Figure 3:**
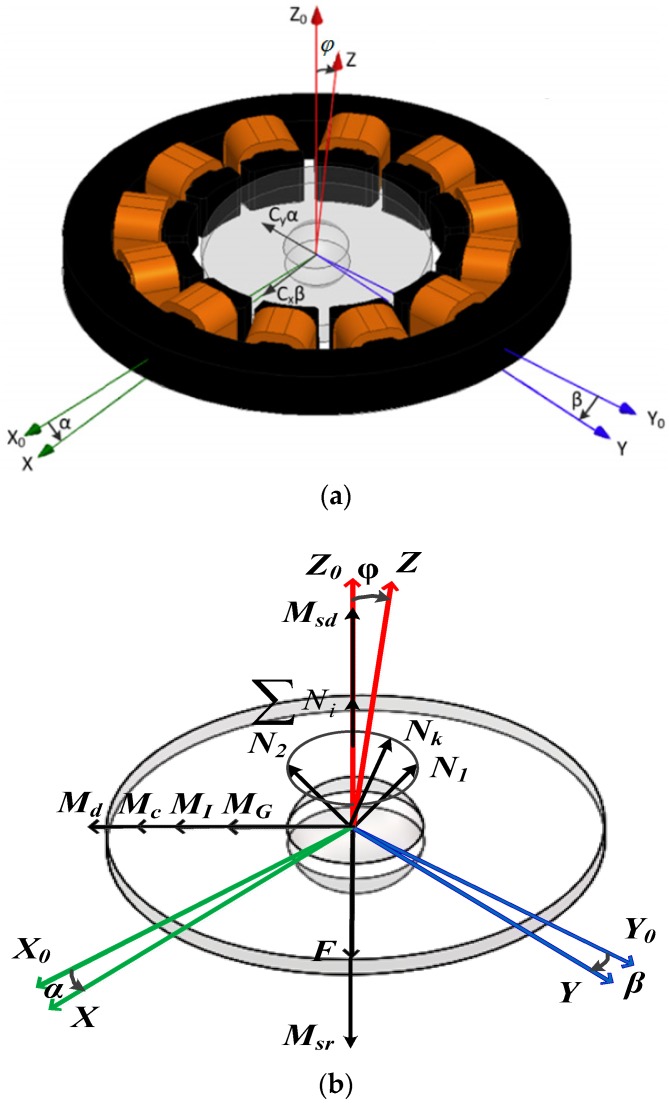
(**a**) operational principle schematic diagram; (**b**) force and torque analysis diagram.

When rotor is actuated to rated spinning speed of 10,000 rpm, a water-film is formed and rotor position relative to the stator is fixed by normal support force *N_i_* from the water-film and the support force *F* from the upper supporting pillar ($\sum N_{i}=-F$). Gravity is neglected here. Input angular speed at the direction of the gyroscope stator plane will produce a proportional Coriolis torque, which deflects the rotor disk from the stator plane. When the rotor is deflected, a magnetic self-restoring torque *M_c_* produces, having the tendency of dragging the rotor disk back to the stator plane. During the rotor precessional motion, a damping torque *M_d_* appears at the interface between the rotor ball and the water-film bearing. Rotor disk deflects under Coriolis inertial torque *M_G_*, self-restoring torque *M_c_*, damping torque *M_d_*, and inertial torque *M_I_*. Rotor disk will be balanced at a certain position with a deflection angle of *φ*, which is small in value for the large self-restoring coefficient. Differential tilt induced capacitance pairs detect deflection angles *α*, *β* at two perpendicular directions (Figure 3a). A force and torque analysis diagram is shown as Figure 3b, in which *M_sr_* and *M_sd_* represent actuating torque and damping torque, respectively. Close-loop driving scheme adopted ensures a stable spinning motion of the rotor during precessional motion, namely, ensuring *M_sr_* equals *M_sd_*.

### 3.1. Self-Restoring Effect of Rotor

**Figure 4:**
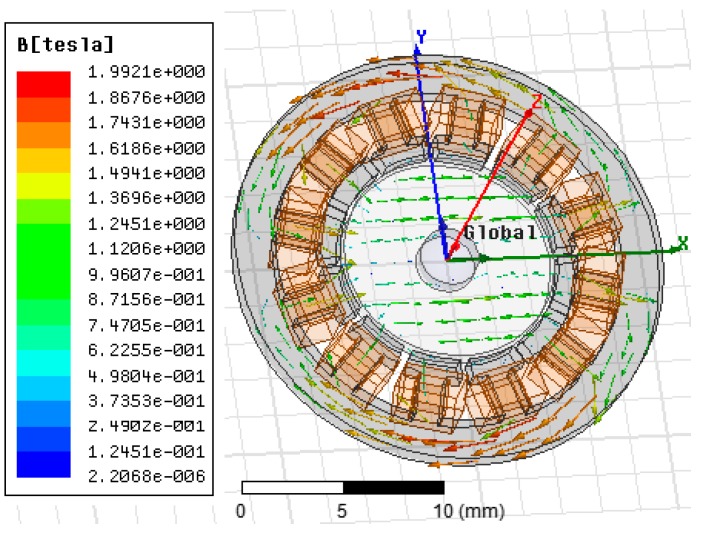
Distribution of magnetic flux density *B*.

The distribution of magnetic flux density *B* from the rotor at the balanced position is shown in Figure 4. Relative magnetic permeability of the magnetic material 2J85 at around 1 T is 1034, and reluctance in air gap is approximately 50 times of that in rotor disk. Then, magnetic energy is mainly stored in the air gap. For isotropic media of air, volume density of magnetic energy is expressed as:

(1) $$\omega_{m}=\frac{1}{2\mu }B^{2}.$$

According to principle of magnetic circuit, magnetic flux lines go through the shortest way between the rotor and the stator. Under rotor deflection angle of *φ* (*δ* = 90°, *δ* is defined as the angle between the rotor deflection axis direction and the rotor magnetization direction), and magnetic energy stored in the air gap between a stator pole and the rotor is calculated as:

(2) $$W_{m}=\frac{B^{2}}{2\mu_{0}}Sg=\frac{B^{2}}{2\mu_{0}}S\sqrt{{\left(R-r\cos\phi \right)}^{2}+{\left(r\sin\phi \right)}^{2}},$$

where *B* is the average magnetic flux density, *μ*_0_ is the permeability of vacuum, *S* is the effective overlap area of the magnetic flux to the stator pole, which is a function of *δ*, *g* is the gap between the rotor disk and the stator pole, *r*, *R* are outer radius of the rotor disk and inner radius of the stator pole. Then, total stored magnetic energy in the gap is *AW_m_*, where *A* is a parameter related to dimensions. Thus, restoring torque *M_c_* is calculated as:

(3) $$\begin{array}{ll} M_{c} & =W_{m}\prime (\phi )=A\frac{B^{2}}{2\mu_{0}}S{\left(R^{2}+r^{2}-2Rr\cos\phi \right)}^{-0.5}Rr\sin\phi \\ & \approx \;A\frac{B^{2}}{2\mu_{0}}S\frac{Rr}{R-r}\phi . \end{array}$$

**Figure 5:**
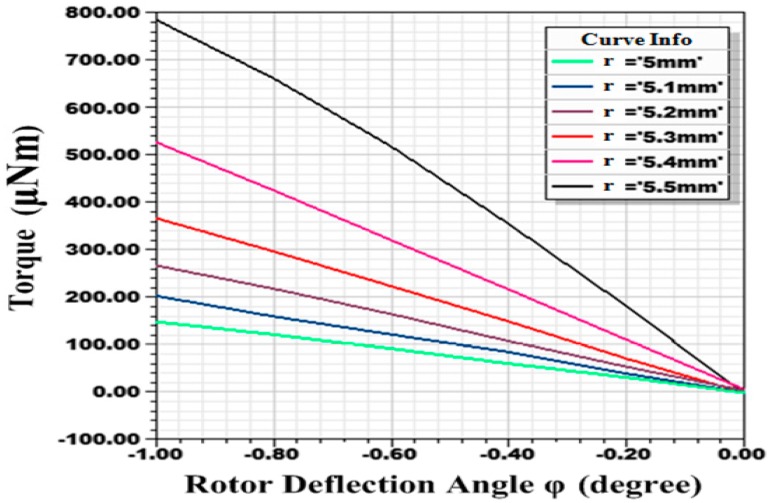
Restoring torques with different radius (*r* values) under deflection angles from −1° to 0°.

**Figure 6:**
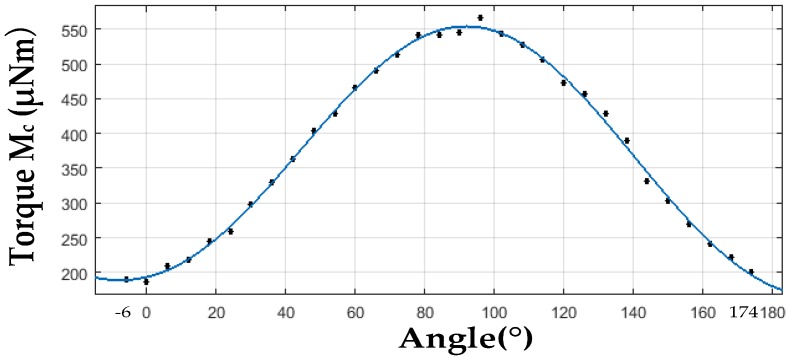
Magnetic self-restoring torque *M_c_* for different *δ* (*φ* = 1°).

It reveals that restoring torque *M_c_* (*δ* = 90°) is proportional to the rotor deflection angle *φ* under the condition that *φ* is of small value (sinϕ≈ϕ, cosϕ≈1) with the coefficient $C=A\frac{B^{2}}{2\mu_{0}}S\frac{Rr}{R-r}$. Therefore, under certain parameters of the stator, increasing rotor radius *r* and improving magnetic flux density *B* will result in a larger restoring coefficient *C*. Rotor is highly magnetized parallel to around 1 tesla at the rotor plane. A stronger magnetic field will cause the rotor disk to be attracted tightly to the stator pole, which prevents the rotor from stable driving. In addition, tight adhesion between the rotor ball and the ball bowl will destroy the superhydrophobic surface on the rotor ball. Restoring torques with different radius (*r* values) under deflection angles from −1° to 0° are simulated in ANSYS Maxwell (16.0, Ansys, Canonsburg, PA, USA) (Figure 5). It can be seen that restoring coefficient *C* increases with the rotor radius *r*, while, when r increases from 5.4 mm to 5.5 mm, linearity deteriorates. Rotor radius is designed as 5.4 mm finally. Because of spinning motion of the magnetic rotor, coefficient *C* is a periodic function of *δ* with the period of 180°. Magnetic self-restoring torque *M_c_*, under rotor deflection angle *φ* of 1°, for different *δ* with the interval of 6° is simulated in ANSYS Maxwell. Output data is put in Matlab (R2016a, MathWorks, Natick, MA, USA) and fitted by a method of sum of sine (Figure 6). Expression of the fitted curve is:

(4) $$\begin{array}{ll} M_{c} & =409.1\sin(0.006942\delta +0.9741)+145.4\sin(0.03484\delta +4.633)\\ & =\;382.86+145.4\sin(0.03484\delta +4.633) \end{array},$$

in which $\frac{1}{180}\int_{0}^{180}409.1\sin(0.006942\delta +0.9741)d\delta =382.86$. The period of *M_c_* (*T* = 2*π*/0.03484 = 180.3) is approximately equal to 180, and maximum, minimum *M_c_* are obtained when rotor deflection axis is perpendicular (*δ* = 90°) and parallel (*δ* = 0°) to magnetization direction. For the proposed gyroscope, angle value (0.03484*δ* + 4.633) can be expressed as (4πft+ψ), where *f* is the frequency of rotor spinning motion, *t* is the time, and *Ψ* is the phase difference between initial state and maximum restoring torque state. Thus, self-restoring coefficient *C* is a period function with the period of 1/2f, expressed as: (382.86 + 145.4 sin (4*πft* + *Ψ*)) μNm/°, namely, (0.0219 + 0.0083 sin (4*πft* + *Ψ*)) (Nm/rad).

### 3.2. Dragging Torque of Water-Film Bearing to Spinning and Precessional Motion

According to the slip model of viscous fluids by Spikes [32], tangential stress *τ_s_* between the water film and the SHS of the rotor is expressed as:

(5) $$\tau_{s}=\tau_{L}+\frac{\eta }{b}v_{s}\approx \frac{\eta }{b}v_{s},$$

where *τ_L_* is the tangential stress limit, which is small in amplitude and omitted, *η* is the viscosity of the water film, *b* is the average slip length, and *v_s_* is the slip speed. Thus, during spinning motion, drag torque *T_fs_* exerted by the water-film bearing on the rotor ball is calculated as:

(6) $$T_{fs}=\int_{S}\tau_{s}(\theta )R_{r}(\theta )ds=2\pi R_{r}^{3}\int_{\gamma }^{r+\kappa }\tau_{s}(\theta ){\left(\cos\theta \right)}^{2}d\theta ,$$

where *γ*, *κ* are marked in Figure 1a, *R_r_*(*θ*) is the distance from the integral position to axis of spinning motion, and *R_r_* is the radius of the rotor ball. *τ_s_* is related to integral position, which is a function of *θ*. SHS turns the contact with the water film to a Cassie state with air bubbles within nanosheets between solid–liquid interface, thus decreasing integral area *S* in (6) and reducing drag torque *T_fs_*. During precessional motion, damping torque *M_d_* exerted on the rotor ball by the water-film bearing is calculated as:

(7) $$M_{d}=\int_{s}\tau_{s1}(\theta )R_{d}(\theta )ds=(\frac{\eta }{b}\int_{s}R_{d}^{2}(\theta )ds)\omega_{p},$$

where *τ_s_*_1_ represents tangential stress during precessional motion, and *R_d_*(*θ*) is the distance from the integral position to axis of precessional motion, both of which are functions of *θ*, *ω_p_* is the precessional angular speed. Value of the expression in brackets is constant for the gyroscope and, thus, damping torque *M_d_* is proportional to *ω_p_*.

### 3.3. Sensing Principle

As shown in Figure 3a, stator and rotor are fixed to *X*_0_*Y*_0_*Z*_0_ and *XYZ* coordinate (without spin motion). For the rated spinning speed is as high as 10,000 rpm, nutation, which is proportional to angular momentum *H* in frequency and inversely proportional to angular momentum *H* in amplitude, is neglected. Thus, inertial torque of rotational motion *M_I_* is neglected. Then, according to force and torque analysis diagram of Figure 3b, gyroscope dynamic balance equations in *X*_0_, *Y*_0_ directions are as below:

(8) $$M_{Gy}+M_{cy}+M_{dy}=(H\omega_{x}-H\dot{\beta })-C_{y}\alpha -D_{y}\dot{\alpha }=0,$$

(9) $$M_{Gx}+M_{cx}+M_{dx}=(-H\omega_{y}-H\dot{\alpha })+C_{x}\beta +D_{x}\dot{\beta }=0,$$

in which, *C_x(y)_*, *D_x(y)_*, ω*_x(y)_* are the electromagnetic elastic coefficient, damping coefficient, input angular speeds in *X*_0_ (*Y*_0_) directions, respectively, and deflection angles *α*, *β* are of small values. For symmetrical structure of the gyroscope, let *C* = *C_x_* = *C_y_*, *D* = *D_x_* = *D_y_*. When *ω_x_*, *ω_y_* are step inputs with amplitude of *A_x_*, *A_y_*, the solutions of Labels (8) and (9) give:

(10) $$\alpha (t)=\frac{H}{C}A_{x}+\frac{H}{C}\sqrt{A_{x}{}^{2}+A_{y}{}^{2}}e^{-\frac{CD}{H^{2}+D^{2}}t}\sin(\frac{\mathrm{CH}}{H^{2}+D^{2}}t+\varphi +\pi ),$$

(11) $$\beta (t)=\frac{H}{C}A_{y}+\frac{H}{C}\sqrt{A_{x}{}^{2}+A_{y}{}^{2}}e^{-\frac{CD}{H^{2}+D^{2}}t}\cos(\frac{CH}{H^{2}+D^{2}}t+\varphi +\pi ),$$

where *φ* = *arctan*(*A_x_/A_y_*). Equations (10) and (11) indicate that the rotor axis will rotate from the null position (where *α* = 0, *β* = 0) at the angular speed of *CH/*(*H*^2^ + *D*^2^) and converge with the time constant of $(H^{2}+D^{2})/CD$ to a static position with deflection angles of *α* = (*H/C*)*A_x_* and *β* = (*H/C*)*A_y_.* As reasoned in Section 3.1, self-restoring coefficient *C* is a function *C*(*t*) expressed in the form of *C*_1_ + *C*_2_ sin (4*πft* + *Ψ*) (*C*_1_ = 0.0219, *C*_2_ = 0.0083). Thus, Equations (10) and (11) are modified as:

(12) $$\alpha (t)=\frac{H}{C(t)}A_{x}+\frac{H}{C(t)}\sqrt{A_{x}{}^{2}+A_{y}{}^{2}}e^{-\frac{D}{H^{2}+D^{2}}\int_{0}^{t}C(t)dt}\sin(\frac{H}{H^{2}+D^{2}}\int_{0}^{t}C(t)dt+\varphi +\pi ),$$

(13) $$\beta (t)=\frac{H}{C(t)}A_{y}+\frac{H}{C(t)}\sqrt{A_{x}{}^{2}+A_{y}{}^{2}}e^{-\frac{D}{H^{2}+D^{2}}\int_{0}^{t}C(t)dt}\cos(\frac{H}{H^{2}+D^{2}}\int_{0}^{t}C(t)dt+\varphi +\pi ).$$

In solutions (12) and (13), $\int_{0}^{t}C(t)dt$ is calculated as:

(14) $$\begin{array}{ll} \int_{0}^{t}C(t)dt & =\int_{0}^{t}C_{1}+C_{2}\sin(4\pi ft+\psi )dt\\ & =C_{1}t+C_{2}\int_{0}^{t}\sin(4\pi ft+\psi )dt\\ & =C_{1}t+\frac{C_{2}}{4\pi f}\left[\cos\psi -\cos(4\pi ft+\psi )\right]. \end{array}$$

In Equation (14), |cosψ−cos(4πft+ψ)|≤2, *C*_2_ is 0.0083 and *f* is 167 in the design. Thus,

(15) $$\begin{array}{l} \left|\int_{0}^{t}C(t)dt-C_{1}t\right|\leq \frac{C_{2}}{4\pi f}\left|\cos\psi -\cos(4\pi ft+\psi )\right|\approx 0\\ \Rightarrow \int_{0}^{t}C(t)dt\approx C_{1}t \end{array}.$$

Then, Equations (10) and (11) are further modified as:

(16) $$\alpha (t)=\frac{H}{C(t)}A_{x}+\frac{H}{C(t)}\sqrt{A_{x}{}^{2}+A_{y}{}^{2}}e^{-\frac{C_{1}D}{H^{2}+D^{2}}t}\sin(\frac{C_{1}H}{H^{2}+D^{2}}t+\varphi +\pi ),$$

(17) $$\beta (t)=\frac{H}{C(t)}A_{y}+\frac{H}{C(t)}\sqrt{A_{x}{}^{2}+A_{y}{}^{2}}e^{-\frac{C_{1}D}{H^{2}+D^{2}}t}\cos(\frac{C_{1}H}{H^{2}+D^{2}}t+\varphi +\pi ).$$

In Equations (16) and (17), $\frac{H}{C(t)}$ can be Taylor expanded under the known condition of $C_{1}>C_{2}$ (*C*_1_ = 0.0219, *C*_2_ = 0.0083) as below:

(18) $$\begin{array}{ll} \frac{H}{C(t)} & =\frac{H}{C_{1}+C_{2}\sin(4\pi ft+\psi )}=\frac{1}{C_{1}}\frac{H}{1-(-\frac{C_{2}}{C_{1}})\sin(4\pi ft+\psi )}\\ & =\;\frac{H}{C_{1}}(1+(-\frac{C_{2}}{C_{1}})\sin(4\pi ft+\psi )+{\left(-\frac{C_{2}}{C_{1}}\right)}^{2}\sin^{2}(4\pi ft+\psi )+o[{\left(-\frac{C_{2}}{C_{1}}\right)}^{2}\sin^{2}(4\pi ft+\psi )])\\ & \approx \;\frac{H}{C_{1}}(1+(-\frac{C_{2}}{C_{1}})\sin(4\pi ft+\psi )) \end{array}.$$

For $C_{1}>C_{2}$, amplitude of DC signal is larger than that of AC signal ($\left|\frac{H}{C_{1}}\right|>\left|\frac{H}{C_{1}}\times \left(-\frac{C_{2}}{C_{1}}\right)\right|$). In conclusion, compared with constant restoring coefficient, sinusoidal self restoring coefficient *C* has little influence on dynamic process of precession; however, it will cause a harmonic motion at a frequency of *2f.* When *ω_x_*, *ω_y_* are impulse inputs with amplitude of *A_x_*, *A_y_*, the solutions of (8) and (9) give:

(19) $$\alpha (t)=\frac{H\sqrt{A_{x}^{2}+A_{y}^{2}}}{\sqrt{H^{2}+D^{2}}}e^{-\frac{C_{1}D}{H^{2}+D^{2}}t}\sin(\frac{C_{1}H}{H^{2}+D^{2}}t+\varphi +\psi +\frac{3\pi }{2}),$$

(20) $$\beta (t)=\frac{H\sqrt{A_{x}^{2}+A_{y}^{2}}}{\sqrt{H^{2}+D^{2}}}e^{-\frac{C_{1}D}{H^{2}+D^{2}}t}\cos(\frac{C_{1}H}{H^{2}+D^{2}}t+\varphi +\psi +\frac{3\pi }{2}),$$

where *ψ = arctan* (*D/H*). Equations (19) and (20) indicate that the rotor axis will rotate from the position with deflection angles of $\alpha (0)=(H\sqrt{A_{x}^{2}+A_{y}^{2}}/\sqrt{H^{2}+D^{2}})\sin(\varphi +\psi +3\pi /2)$ and $\beta (0)=(H\sqrt{A_{x}^{2}+A_{y}^{2}}/\sqrt{H^{2}+D^{2}})\cos(\varphi +\psi +3\pi /2)$ to the null position at the angular speed of *C*_1_*H/*(*H*^2^ + *D*^2^). Sinusoidal self restoring coefficient *C* has no influence on impulse response.

### 3.4. Differential Capacitance Detection and Signal Processing

**Figure 7:**
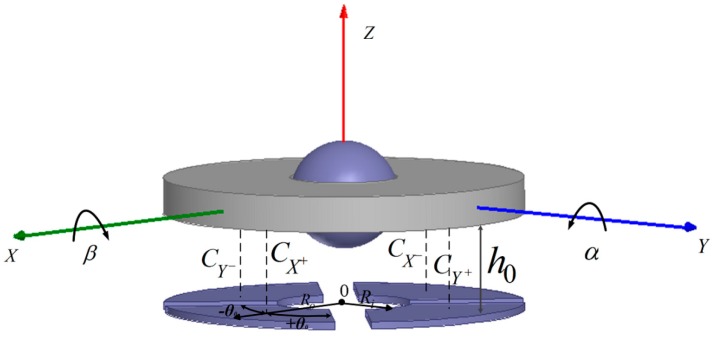
Schematic diagram of differential pairs testing *α* and *β*.

**Figure 8:**
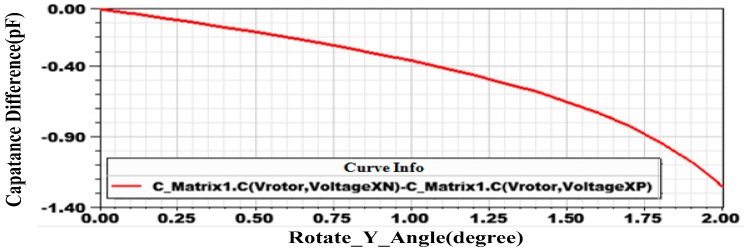
Capacitance difference for different deflection angles.

As stated in Section 3.3, angles of *α* and *β* reveal input angular speeds in *X*_0_ and *Y*_0_ directions. Differential capacitance detection is applied. Detection electrode plate with four poles and the rotor disk form four tilt induced capacitors, with radially opposite ones forming a differential pair. Schematic diagram of two differential pairs, $C_{X^{+}}$ and $C_{X^{-}}$, $C_{Y^{+}}$ and $C_{Y^{-}}$, testing angles of *α* and *β* are shown in Figure 7. Four initial capacitances with no angular speed inputs are $C_{X_{0}^{+}}$, $C_{X_{0}^{-}}$, $C_{Y_{0}^{+}}$, and $C_{Y_{0}^{-}}$. Four capacitances are approximately the same and main errors come from fabrication. For the symmetry of the structure, one differential pair of $C_{X^{+}}$ and $C_{X^{-}}$ is analyzed here. According to [33],

(21) $$C_{X^{+}}=C_{X_{0}^{+}}+\frac{2\alpha \varepsilon (R_{o}^{3}-R_{i}^{3})\sin\theta_{0}}{3h_{0}^{2}}+o(\alpha ,\beta ),$$

(22) $$C_{X^{-}}=C_{X_{0}^{-}}-\frac{2\alpha \varepsilon (R_{o}^{3}-R_{i}^{3})\sin\theta_{0}}{3h_{0}^{2}}+o(\alpha ,\beta ),$$

when *α*, *β* are deflection angles of small values. Capacitance differences under deflection angle from 0° to 2° are simulated in Maxwell as Figure 8. In the simulation, $C_{X_{0}^{+}}$ and $C_{X_{0}^{-}}$ are the same and according to Labels (21) and (22), $\Delta C=C_{X^{+}}-C_{X^{-}}=\frac{4\alpha \varepsilon (R_{o}^{3}-R_{i}^{3})\sin\theta_{0}}{3h_{0}^{2}}.$ As can be seen (Figure 8), when the deflection angle is within 1°, capacitance difference is roughly proportional to the rotor deflection angle, which restricts the measurement range of the gyroscope.

**Figure 9:**
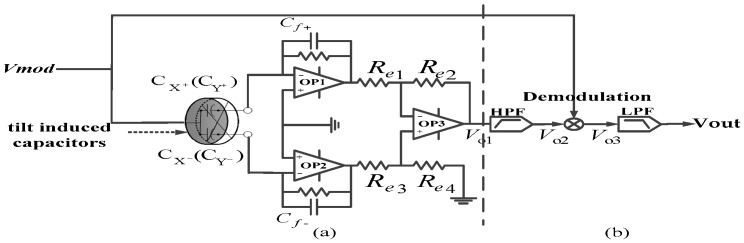
Signal processing system: (**a**) signal detection part; (**b**) signal filtering part.

To detect the capacitance change, AC modulation signal *V_mod_* (2 V, 15 kHz) is added to the upper surface of the rotor ball. Currents go through the tilt induced capacitor pair ($C_{X^{+}}$, $C_{X^{-}}$), differential amplifier, proportional amplifier and obtain the output voltage *V_o_*_1_ as shown in Figure 9a. Tilt induced capacitors *C_f+_*, *C_f−_* are adjusted to satisfy:

(23) $$\frac{C_{X_{0}^{+}}}{C_{f+}}=\frac{C_{X_{0}^{-}}}{C_{f-}},$$

so that *V*_o1_ at initial state (with no angular speed input) is zero. The amplification factor of the proportional amplifier is adjusted to be *G_op3_*, and then the output of *V*_o1_ is expressed as:

(24) $$V_{o1}=G_{op3}V_{\mathrm{mod}}(\frac{C_{X^{+}}}{C_{f+}}-\frac{C_{X^{-}}}{C_{f-}}).$$

Taking Labels (21)–(23) into Label (24), the following expression is obtained:

(25) $$V_{o1}=G_{op3}V_{\mathrm{mod}}(\frac{1}{C_{f+}^{}}+\frac{1}{C_{f-}^{}})\frac{2\varepsilon (R_{0}^{3}-R_{i}^{3})\sin\theta_{0}}{3h_{0}^{2}}\alpha .$$

It can be seen from Label (25) that the amplitude of AC voltage *V*_o1_ is proportional to deflecting angle *α*. Similarly, the amplitude of the output voltage of the other differential pair $C_{Y^{+}}$ and $C_{Y^{-}}$ is proportional to deflecting angle *β*. Differential signal *V*_o1_ is then processed as Figure 9b. DC component in the signal is removed by high-pass filter first and then the signal is demodulated by multiplying modulation signal *V_mod_*. Finally, low-pass filter, with cut-off frequency of 10 Hz, is added to obtain low-frequency output voltage signal.

## 4. Measurement and Discussion

**Figure 10:**
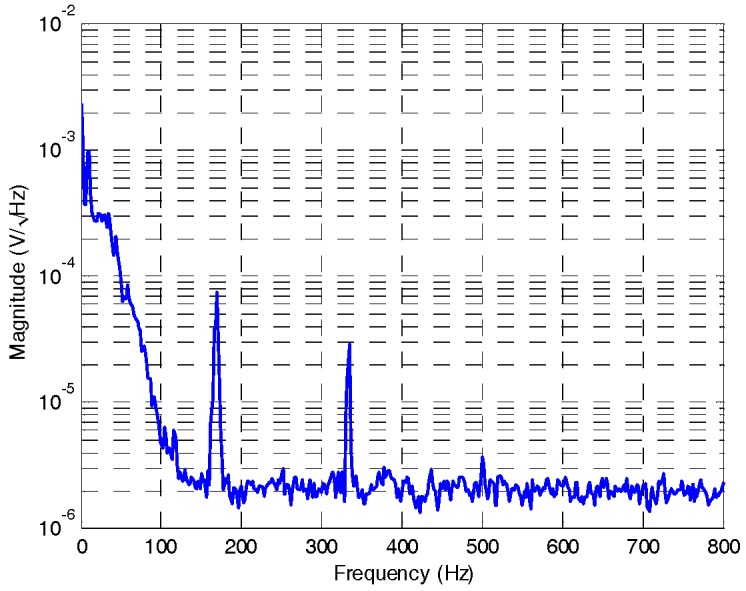
Spectral density of output signal.

Before setting of the cut-off frequency of LPF (Figure 9), a 50 Hz LPF is applied and output signal is tested by spectral analyzer as Figure 10, in which, precession frequency (12 Hz) signal component, 1/f noise, noise components of driving frequency (166.7 Hz) and two-fold of driving frequency (333 Hz), can be seen apparently. In addition, 167 Hz and 333 Hz noise components derive from structural asymmetry brought by fabrication error and periodic rotor self-restoring effect, respectively. Cut-off frequency of LPF is set as 10 Hz to filter noises, finally. A lower cut-off frequency will result in a longer output lag.

**Figure 11:**
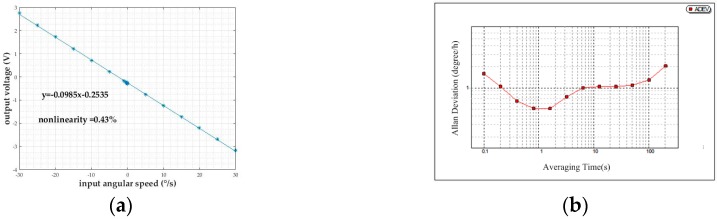
(**a**) linear fitting of output voltages under input angular speed range of −30°/s to 30°/s; (**b**) log-log plot of Allan deviation versus averaging time.

Sensitivity and nonlinearity of the proposed gyroscope are measured by linear fitting (with Matlab) of output voltages under evenly-distributed (with 5°/s intervals) input angular speeds from −30°/s to 30°/s (Figure 11a). Resolution is obtained by analysis of responses to nine input angular speeds, which are exponentially-distributed from −0.00625°/s to −1.6°/s (noted in Figure 11a). Low frequency sensitivity, nonlinearity within input angular speed range of −30°/s to 30°/s (limited by linearity of differential capacitance detection), and resolution is confirmed as 0.0985 V/(°/s), 0.43%, 0.1°/s, respectively. For bias stability test, no input angular speed is given, and output voltages over the time span of 0.5 h are recorded and processed. Then, bias stability is confirmed by an Allan deviation curve (lowest value of Allan deviation) to be 0.5°/h (Figure 11b).

**Figure 12:**
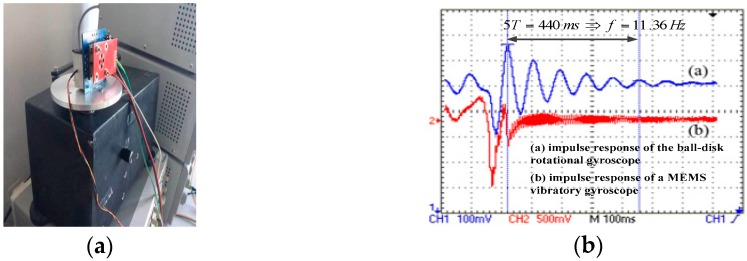
(**a**) photograph of the rate table with the proposed rotational gyroscope and a Micro-electromechanical Systems (MEMS) quartz vibratory gyroscope on it for dynamic characteristic test; (**b**) impulse responses of the proposed rotational gyroscope and a MEMS quartz vibratory gyroscope.

To test dynamic characteristics of the proposed gyroscope and verify its functionality, the proposed rotational gyroscope and a MEMS quartz vibratory gyroscope are placed on the oscillating platform as shown in Figure 12a. An impulse angular speed is given by the oscillating platform and impulse responses of two gyroscopes are shown in Figure 12b. Similar response curves verify the functionality of the proposed gyroscope. Curve (a) is fitted in the form of (19) and parameters of *C*, *D*, H are identified to be 0.02017, 2.971 × 10^−5^, respectively, with calculated *H* of 2.784 × 10^−4^. Lower amplitude of *C* than theoretical value of 0.0219 calculated in Section 3.1 is because of factors such as manufacture error and magnetic flux leakage.

## 5. Conclusions

A rotational gyroscope with a ball-disk shaped rotor supported by a water-film bearing and actuated by the driving scheme of BLDCM is proposed in the paper. The rotor disk is magnetized in a parallel direction. When an input angular speed acts on the gyroscope and the rotor deflects under Coriolis torque, magnetic energy stored in the gap is increased and thus a restoring torque *M_c_*, proportional to rotor deflection angle *φ*, is produced, which balances Coriolis torque. With spinning motion of the rotor, *M_c_* changes in sinusoidal waveform, and the average restoring coefficient is 0.02017 Nm/rad. Water-film bearing adopted in the design, as a substitute to complicated rotor suspension system in MSGs and ESGs, provides stable rotor support with low friction. To further decrease sliding friction, an SHS with water CA of 167° is fabricated on the rotor ball, which increases rated spinning speed from 8970 rpm to 10,084 rpm. Differential capacitance detection is adopted by installing four sectorial poles on a plate under the rotor disk. Simulation by Maxwell indicates that detection linearity is ideal when rotor deflection angle is under 1°, which limits measurement range of the proposed gyroscope to −30°/s–30°/s. Spectral analysis to output voltage signal was done to confirm cut-off frequency of LPF and 333 Hz noise component was observed, which is produced by magnetic self-restoring effect of the rotor. Static measurement parameters and dynamic parameters are acquired through experiments, with the low bias stability of only 0.5°/h. Excellent performance is the result of the special structure design with a water-film bearing together with the utilization of magnetic self-restoring effect of the rotor.
